# Recurrent Severe Viral-Induced Rhabdomyolysis Associated With Underlying Genetic Variants in a Young Adult: A Case Report

**DOI:** 10.7759/cureus.114691

**Published:** 2026-08-17

**Authors:** Alexandra M Stone, Connor M Bunch, Andrew Gauger, Glenn Seela, Nadia Aboumourad, Jordan Maust, Alexandra Picardal, Creed J Roschyk, Brendan A Gonzales, Avery C Dollard, Amro Abdelghani, Brandtly Yakey, Robert G Klever, Joseph Miller

**Affiliations:** 1 Emergency Medicine, Wayne State University School of Medicine, Detroit, USA; 2 Emergency Medicine, Henry Ford Health System, Detroit, USA; 3 Internal Medicine, Henry Ford Health System, Detroit, USA; 4 Critical Care Medicine, Henry Ford Health System, Detroit, USA; 5 Dermatology, Henry Ford Health System, Detroit, USA; 6 Radiology, Henry Ford Health System, Detroit, USA; 7 Nephrology, Henry Ford Health System, Detroit, USA

**Keywords:** acute kidney injury, case report, genetic testing, influenza a virus, rhabdomyolysis

## Abstract

Rhabdomyolysis is a potentially life-threatening syndrome characterized by skeletal muscle breakdown and release of intracellular contents into circulation, which can result in electrolyte abnormalities, acute kidney injury (AKI), and renal failure. Viral infections, particularly influenza, are recognized triggers of rhabdomyolysis; however, recurrent or unusually severe presentations may suggest underlying genetic susceptibility. We report a case of a previously healthy 21-year-old male with a prior history of COVID-19-associated rhabdomyolysis who presented to the emergency department with fever, generalized weakness, myalgia, red urine, and decreased oral intake following influenza A infection. Laboratory evaluation demonstrated severe rhabdomyolysis with creatine kinase (CK) elevation from 512,650 IU/L to a peak of over 1,000,000 IU/L, complicated by pigment-induced AKI requiring temporary hemodialysis. Extensive autoimmune, metabolic, and neuromuscular evaluation was unremarkable. Subsequent genetic testing identified a heterozygous pathogenic STAC3 variant and variants of uncertain significance in SCN4A, PLEC, and MYH7, raising suspicion for an underlying inherited predisposition to recurrent viral-induced muscle injury. This case highlights the importance of considering genetic susceptibility in patients with recurrent or severe viral-induced rhabdomyolysis, particularly in the setting of extreme CK elevation and recurrent episodes.

## Introduction

Rhabdomyolysis is a potentially life-threatening condition characterized by the breakdown of skeletal muscle and the release of intracellular muscle contents, such as myoglobin, creatine kinase (CK), and lactate dehydrogenase, into circulation [1]. Clinical manifestations range from asymptomatic illness with isolated serum CK elevation to a clinical syndrome of myalgia, muscle weakness and swelling, and myoglobinuria, with extreme elevations in CK levels, often leading to acute renal failure [2].

Common triggers include trauma, strenuous physical activity, infections, genetic disorders, ischemia, drugs, toxins, and metabolic disorders. Viral infections, including influenza viruses, coxsackieviruses, Epstein-Barr virus, herpes simplex virus, parainfluenza viruses, and adenoviruses, have also been implicated in the development of rhabdomyolysis [3]. While genetically susceptible patients are at a particularly increased risk for nontraumatic rhabdomyolysis, the interplay between viral triggers and genetic susceptibility remains incompletely understood.

We report a case of a 21-year-old male with recurrent, severe, viral-induced rhabdomyolysis associated with extreme CK elevations, where further evaluation revealed genetic variants raising suspicion for an underlying predisposition to recurrent viral-induced rhabdomyolysis.

## Case presentation

A 21-year-old male presented to the emergency department (ED) with a four-day history of red urine, fever, generalized weakness, pain, and nausea. He reported decreased oral intake and progressively worsening weakness and pain in his bilateral upper and lower extremities. He denied recent trauma or injuries, sick contacts, chest pain, shortness of breath, syncope, palpitations, dysuria, abdominal pain, emesis, hemoptysis, diarrhea, or constipation.

His past medical history was significant for moderate asthma and multiple allergies. He had a prior episode of rhabdomyolysis following a COVID-19 infection at the age of 17. Developmental history was largely unremarkable, with resolved speech delay and prior active participation in sports and physically demanding work. There was no family history of peripheral neuropathy, muscle disease, amyotrophic lateral sclerosis, or metabolic disorders. His recent occupational history included work in a fast food restaurant.

**Figure 1:**
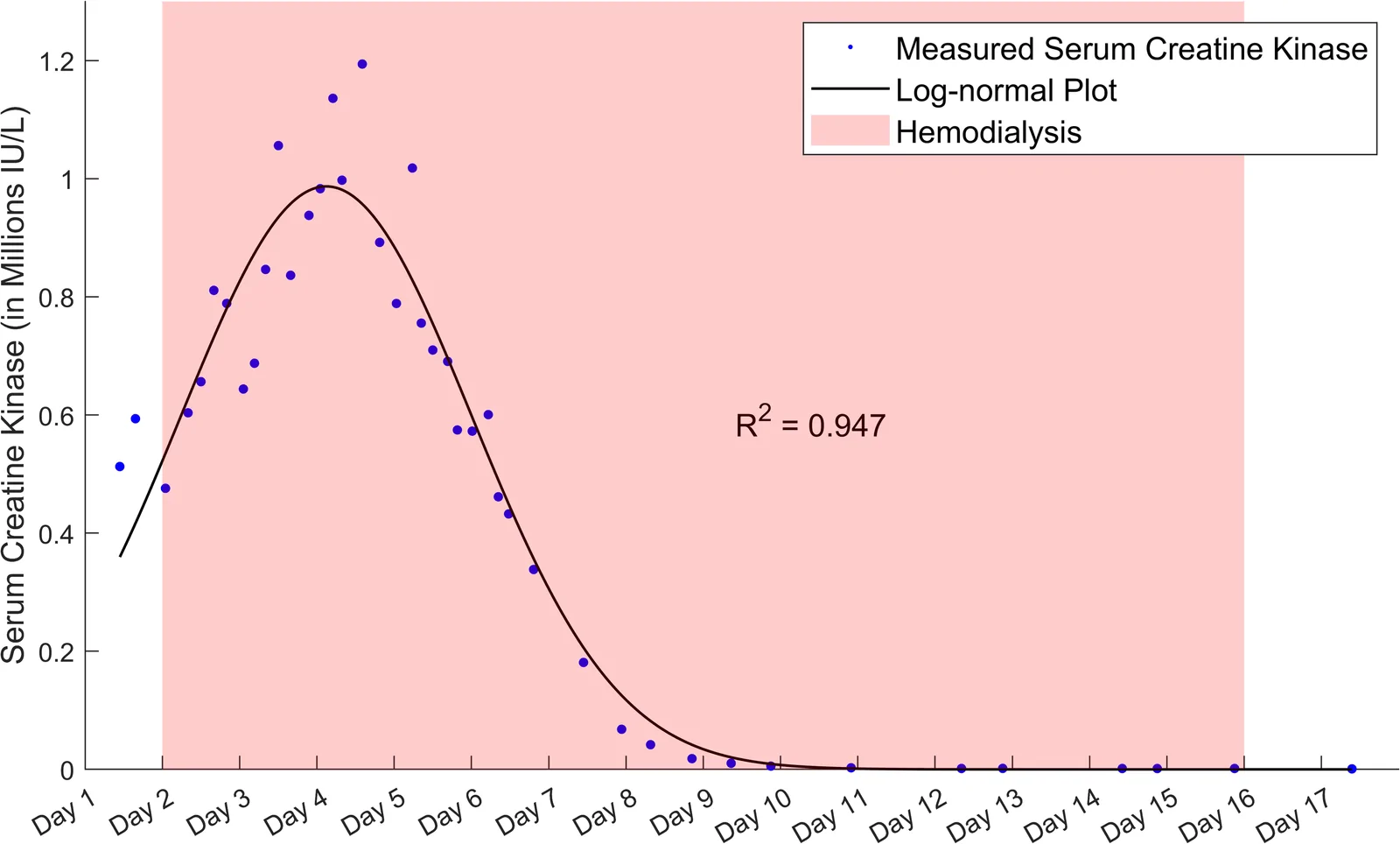
Creatine kinase (CK) levels over the course of hospitalization. These data indicate a serum CK peak around day 4 of hospitalization. The blue dots identify specific CK values recorded at specific times, while the red curve is a three parameter, shifted, log-normal curve, estimated by a nonlinear least-squares regression using an unconstrained simplex search, utilizing the Nelder-Mead simplex algorithm. The duration of hemodialysis is indicated in red.

**Figure 2:**
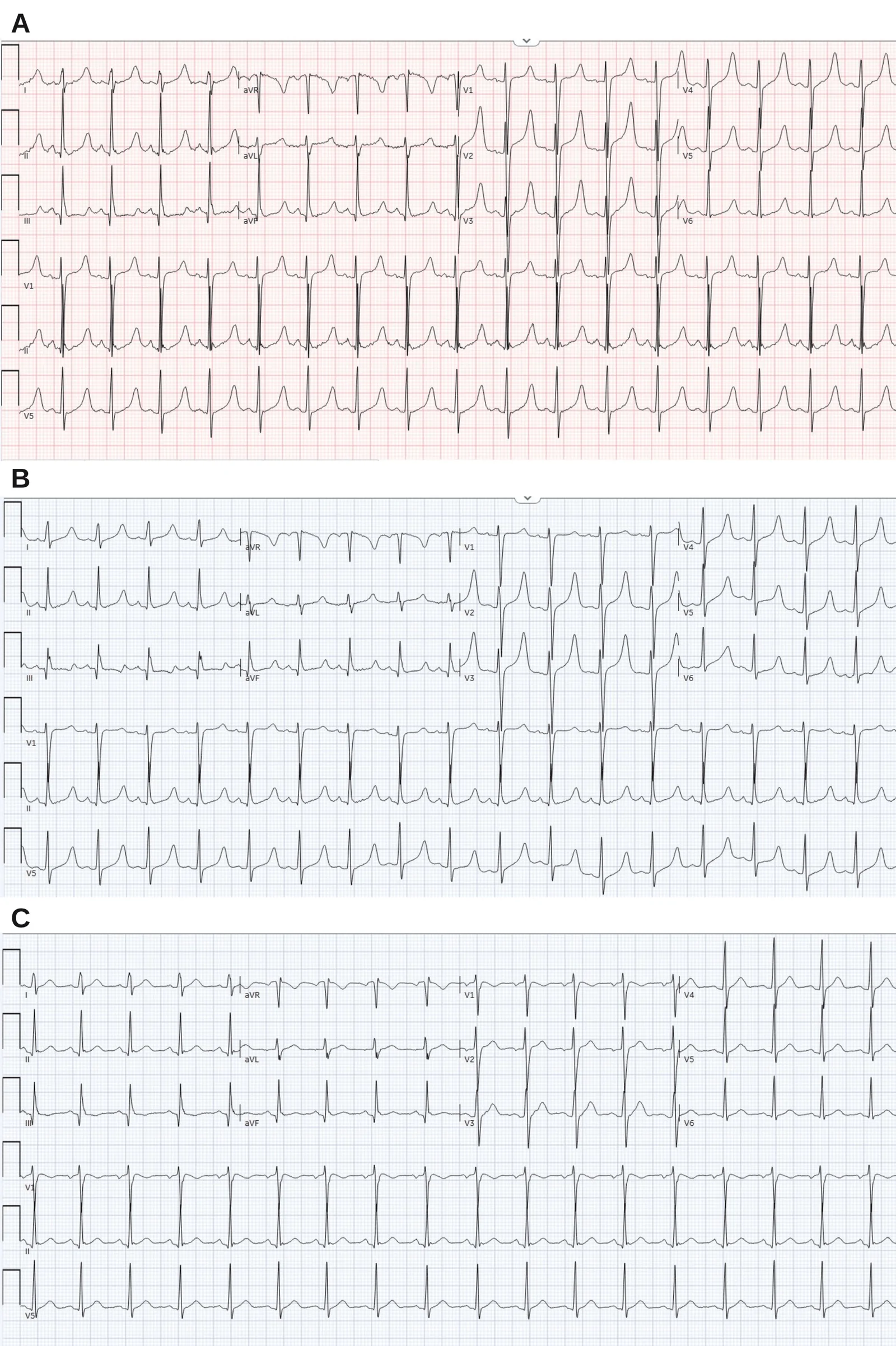
Electrocardiography (ECG) findings from admission to day 8. Figure 2A shows the patient's ECG at presentation, demonstrating sinus tachycardia with a ventricular rate of 106 bpm, a prolonged corrected QT interval of 528 ms, and peaked T waves. Figure 2B shows the patient's ECG on day 1 of admission after receiving their first session of dialysis. Figure 2C shows the last available ECG taken on day 8 of admission.

On arrival to the ED, vital signs were notable for hypertension (143/99 mmHg) and tachycardia (111 beats per minute). Physical exam demonstrated bilateral generalized weakness of the upper and lower extremities. Initial laboratory investigations are summarized in Table 1. Notably, CK rose from 512,000 IU/L to a peak of 1,194,000 IU/L on day 4 of admission (Figure 1). Viral testing was positive for influenza A. Urinalysis revealed brown urine, myoglobinuria (i.e., positive for blood with no RBCs), and positive nitrite, indicating a possible urinary tract infection, as well as glucosuria and ketonuria. A urine drug screen was negative. The peripheral smear showed leukocytosis and normocytic normochromic anemia but was negative for schistocytes, dysplasia, and blasts. Electrocardiography (ECG) showed sinus tachycardia with a ventricular rate of 106 bpm, a prolonged corrected QT interval of 528 ms, and peaked T waves (Figure 2). Chest X-ray results were consistent with viral pneumonia.

**Table 1:** Results of the initial laboratory investigations.

Blood Test	Result	Reference Range
Creatine kinase	512,650	<250 IU/L
Lactate dehydrogenase (LDH)	>6000	<250 IU/L
Magnesium	3.4	1.8–2.3 mg/dL
Phosphorus	12.5	2.5–4.5 mg/dL
Calcium, ionized	0.44	1.0–1.35 mmol/L
Sodium	130	135–145 mmol/L
Potassium	5.5	3.5–5.0 mmol/L
Blood urea nitrogen (BUN)	26	10–25 mg/dL
Creatinine	1.74	<1.3 mg/dL
Calcium	7.0	8.2–10.2 mg/dL
Glomerular filtration rate (GFR)	56	>60 mL/min/1.73m^2^
Hemoglobin	18.1	13.5–17.0 g/dL
White cell count	10.1	3.8–10.6 K/µL
Neutrophil count	7.50	1.80–7.70 K/µL
Platelet count	546	150–450 K/µL
Aspartate aminotransferase (AST)	1547	<45 IU/L
Alanine aminotransferase (ALT)	123	<52 IU/L
Gamma-glutamyl transferase (GGT)	54	0–70 IU/L

He was admitted to the intensive care unit with nontraumatic rhabdomyolysis (secondary to influenza A) complicated by pigment nephropathy with a superimposed prerenal component, hyperkalemia, hyperphosphatemia, and hypocalcemia. He was started on aggressive intravenous fluids, an extended seven-day course of oseltamivir, calcium gluconate for ECG findings, thiamine supplementation, and hydromorphone for pain control. His hyperkalemia was treated with a potassium binder and insulin with D50, and his hyperphosphatemia with a phosphate binder. Given worsening CK and electrolyte derangements, he started hemodialysis on day 2 of admission.

During hospitalization, a comprehensive diagnostic workup was initiated with neurology to determine the cause of the patient’s rhabdomyolysis. Acquired causes of recurrent rhabdomyolysis, including inflammatory and necrotizing myopathies, were ruled out with serologic testing and a myositis antibody panel. Genetic, autoimmune, and metabolic testing for hereditary neuromuscular disorders, including muscular dystrophies, inherited myopathies, mitochondrial disorders, congenital myasthenic syndromes, fatty acid oxidation disorders, glycogen storage diseases, and autoimmune inflammatory myopathy, was unremarkable/negative. Abnormal acylcarnitine levels were initially noted during hospitalization but were normal on repeat testing.

He was discharged home on day 16 with outpatient dialysis and follow-up with neurology for further diagnostic evaluation, where diffuse hyperreflexia was noted. He completed dialysis treatment two weeks after discharge as kidney function gradually returned to baseline, normalizing to 85 IU/L within a month of discharge.

At his first follow-up, genetic testing revealed a heterozygous pathogenic STAC3 variant as well as variants of uncertain significance (VUS) in SCN4A, PLEC, and MYH7 (Table 2). An electromyography (EMG) of the right side was performed, showing no evidence of myopathy or neuropathy. MRI of the brain was negative for any acute intracranial abnormalities. Muscle MRI with dermatomyositis protocol revealed increased swelling in the calf muscles (right greater than left) and the shoulder girdle muscles, suggestive of ongoing muscle injury/inflammation despite clinical recovery. He was subsequently referred to a geneticist for further evaluation of STAC3, SCN4A, and other relevant panels. He has also been referred to neurosurgery for a muscle biopsy of the right calf muscle as a potential next step in the diagnostic workup.

**Table 2:** Results of the comprehensive neuromuscular disorder panel.

Gene	Variant	Zygosity	Variant Classification
STAC3	c.851G>C (p.Trp284Ser)	Heterozygous	Pathogenic
MYH7	c.3152C>T (p.Ala1051Val)	Heterozygous	Uncertain Significance
PLEC	c.3614G>A (p.Arg1205Gln)	Heterozygous	Uncertain Significance
SCN4A	c.2802G>C (p.Glu934Asp)	Heterozygous	Uncertain Significance

Since that time, the patient has resumed daily activities and work and reports feeling back to baseline strength, with no ongoing muscle weakness or new neurological complaints.

## Discussion

This case describes recurrent and severe viral-induced rhabdomyolysis in a previously healthy young adult with extreme CK elevation and underlying genetic variants associated with skeletal muscle dysfunction. Rhabdomyolysis is characterized by skeletal muscle breakdown resulting in the release of intracellular muscle contents, including CK, myoglobin, and electrolytes, into circulation. In the absence of myocardial or cerebral infarction, CK levels greater than 5000 U/L are highly suggestive of significant muscle injury [4]. Our patient demonstrated profound CK elevation exceeding 1 million IU/L, representing one of the highest reported CK levels in viral-induced rhabdomyolysis [5-9].

Viral infections, particularly influenza A and B, are recognized causes of rhabdomyolysis [3, 10]. Proposed mechanisms include direct viral invasion of myocytes, cytokine-mediated muscle injury, and systemic inflammatory responses leading to myonecrosis [9, 10]. Although several cases of influenza-associated rhabdomyolysis have been reported previously, most involve mild-to-moderate elevations in CK and occur in pediatric or immunocompromised patients [9]. In contrast, our patient experienced two distinct episodes of severe rhabdomyolysis, both preceded by viral illnesses, including COVID-19 and influenza A, suggesting an underlying predisposition to muscle injury. During his first episode, he was hospitalized for five days and effectively treated with intravenous fluids without requiring dialysis, unlike in his current episode.

Increasing evidence supports a “two-hit” model in which environmental stressors/triggers, such as infection or exertion, precipitate rhabdomyolysis in genetically susceptible individuals [2, 11, 12]. Patients presenting with recurrent episodes, CK concentrations greater than 50 times the upper limit of normal, persistent hyperckemia, or a family history of neuromuscular disease should prompt consideration of an inherited myopathy or metabolic disorder [11, 13].

Several genetic mutations/conditions have been shown to influence rhabdomyolysis susceptibility, including metabolic myopathies, mitochondrial and neuromuscular disorders, muscular dystrophies, and metabolic diseases (e.g., disorders of lipid and glycogen metabolism) (Table 3) [2, 12, 14]. Although workup for inherited metabolic, autoimmune, and neuromuscular disorders during hospitalization was unremarkable, genetic testing identified variants in several genes associated with hereditary neuromuscular conditions, including STAC3 and SCN4A. 

**Table 3:** Genetic disorders associated with rhabdomyolysis.

Gene	Disorder
AGL	Glycogen storage disease type IIIa
ACADM	Medium-chain acyl-CoA dehydrogenase deficiency
ACADVL	Very-long-chain acyl-CoA dehydrogenase (VLCAD) deficiency
ANO5	Limb-girdle muscular dystrophy type 2L (LGMD2L), Asymptomatic hyperCKemia
CAPN3	Limb-girdle muscular dystrophy Type 2A (LGMD2A)
CNBP	Myotonic dystrophy type II
CPT2	Carnitine palmitoyltransferase II (CPT II) deficiency
DMD	Duchenne muscular dystrophy, Becker muscular dystrophy
DMPK	Myotonic dystrophy type I
DYSF	Limb-girdle muscular dystrophy type 2B
FKRP	Limb-girdle muscular dystrophy type 2I (LGMD2I/R9)
HADHA	Long-chain 3-hydroxyacyl-CoA dehydrogenase deficiency, Mitochondrial trifunctional protein deficiency
LPIN1	Acute recurrent autosomal recessive myoglobinuria, Phosphatidic acid phosphatase deficiency
MAGT1	XMEN disease (X-linked immunodeficiency with magnesium defect, Epstein-Barr virus infection, and neoplasia)
PGM1	Congenital glycosylation disorder type It
PYGM	Glycogen storage disease type V, McArdle disease
RYR1	Malignant hyperthermia-susceptibility, Exertional rhabdomyolysis, Congenital myopathy
SCN4A	Congenital paramyotonia
SCGA	Limb-girdle muscular dystrophy type 2D (LGMD2D)
SMPD1	Niemann‐Pick disease, type A/B
STAC3	Congenital myopathy (Native American myopathy), malignant hyperthermia
TANGO2	Metabolic encephalomyopathy, recurrent rhabdomyolysis, cardiac arrhythmias and neurodegeneration

STAC3 encodes a protein essential for skeletal muscle excitation-contraction coupling through interactions with the ryanodine and dihydropyridine receptor complexes [15]. Pathogenic variants in STAC3 (homozygous pathogenic variants in the STAC3 gene (c.851G > C, p.Trp284Ser) are associated with congenital myopathy (previously known as Native American myopathy) and malignant hyperthermia (MH) susceptibility [13]. Disruption of excitation-contraction coupling may lower the threshold for muscle injury during physiologic stress [16]. Similarly, SCN4A encodes the voltage-gated skeletal muscle sodium channel NaV1.4, which regulates membrane excitability and the generation of skeletal muscle action potentials [17]. Mutations in SCN4A have been linked to a spectrum of neuromuscular disorders, including periodic paralysis, paramyotonia congenita, exercise intolerance, and episodic myopathy [17]. Defects in membrane excitability or sodium ion channel function may increase susceptibility to muscle injury during metabolic or infectious stress [18].

While the precise clinical significance of the STAC3 and SCN4A variants in our patient remains undetermined, their coexistence suggests a possible inherited susceptibility to recurrent rhabdomyolysis. Given the association between STAC3 and SCN4A variants and MH, the patient should be considered potentially susceptible to MH-like reactions to volatile anesthetics pending additional testing and evaluation with a geneticist.

## Conclusions

In conclusion, this case highlights the importance of considering underlying genetic susceptibility in patients with recurrent or unusually severe viral-induced rhabdomyolysis. Infection-triggered rhabdomyolysis may represent the initial manifestation of an inherited muscle disorder, even in patients without overt neuromuscular symptoms or family history. Recognition of this association has important implications for diagnostic evaluation, genetic counseling, recurrence prevention, and future anesthetic risk assessment, particularly in patients with variants associated with MH susceptibility.
